# HbA1C Variability Is Strongly Associated With the Severity of Peripheral Neuropathy in Patients With Type 2 Diabetes

**DOI:** 10.3389/fnins.2019.00090

**Published:** 2019-02-13

**Authors:** Yun-Ru Lai, Wen-Chan Chiu, Chih-Cheng Huang, Nai-Wen Tsai, Hung-Chen Wang, Wei-Che Lin, Ben-Chung Cheng, Yu-Jih Su, Chih-Min Su, Sheng-Yuan Hsiao, Cheng-Hsien Lu

**Affiliations:** ^1^Department of Biological Science, National Sun Yat-Sen University, Kaohsiung, Taiwan; ^2^Department of Neurology, Kaohsiung Chang Gung Memorial Hospital, Chang Gung University College of Medicine, Kaohsiung, Taiwan; ^3^Department of Internal Medicine, Kaohsiung Chang Gung Memorial Hospital, Chang Gung University College of Medicine, Kaohsiung, Taiwan; ^4^Department of Neurosurgery, Kaohsiung Chang Gung Memorial Hospital, Chang Gung University College of Medicine, Kaohsiung, Taiwan; ^5^Department of Radiology, Kaohsiung Chang Gung Memorial Hospital, Chang Gung University College of Medicine, Kaohsiung, Taiwan; ^6^Department of Emergency Medicine, Kaohsiung Chang Gung Memorial Hospital, Chang Gung University College of Medicine, Kaohsiung, Taiwan; ^7^Center for Shockwave Medicine and Tissue Engineering, Kaohsiung Chang Gung Memorial Hospital, Chang Gung University College of Medicine, Kaohsiung, Taiwan; ^8^Department of Neurology, Xiamen Chang Gung Memorial Hospital, Xiamen, China

**Keywords:** composite scores of nerve conduction, diabetic peripheral neuropathy, HbA1C variability, SD-HbA1c, Type 2 diabetes, severity of peripheral neuropathy

## Abstract

Variability in HbA1c is associated with a higher risk of cardiovascular disease and microvascular complications in patients with type 2 diabetes. The present study evaluated the severity of somatic nerve dysfunction at different stages of chronic glycemic impairment, and its correlation with different cardio-metabolic parameters. The study was conducted on 223 patients with type 2 diabetes. We calculated the intrapersonal mean, standard deviation (SD), and coefficient of variation of HbA1c for each patient using all measurements obtained for 3 years prior to the study. Patients were divided into quartiles according to the SD of HbA1c, and we constructed composite scores of nerve conduction as the severity of peripheral neuropathy. Linear regression analysis was performed to evaluate the influence of independent variables on mean composite scores. Those with higher SD-HbA1c had a higher body mass index, mean and index HbA1c, triglyceride and uric acid level, urinary albumin excretion and albumin-creatinine ratio, proportion of insulin therapy, and prevalence of hypertension as the underlying diseases, but lower estimate glomerular filtration rate (eGFR). In addition, those with higher SD-HbA1c showed lower amplitudes and reduced motor nerve conduction velocity in tested nerves, and lower sensory nerve conduction velocity in the sural nerve. Furthermore, those with higher SD-HbA1c had higher composite scores of low extremities. Multiple linear regression analysis revealed that diabetes duration, SD-HbA1c, and eGFR were independently associated with mean composite scores. Based on our results, HbA1c variability plus chronic glycemic impairment is strongly associated with the severity of peripheral neuropathy in patients with type 2 diabetes. Aggressively control blood glucose to an acceptable range and avoid blood glucose fluctuations by individualized treatment to prevent further nerve damage.

## Introduction

Diabetic peripheral neuropathy (DPN), including autonomic and somatic neuropathy, is a very important microvascular complication of diabetes. The most common somatic neuropathy, diabetic sensorimotor polyneuropathy (DSPN), is an important cause of foot ulcer and amputation in diabetic patients that can result in delayed diagnosis due to a protracted asymptomatic course (Boulton et al., 2005). Moreover, the clinical manifestations of DSPN are broad, and often are not consistent with objective measurement results.

It is not sufficient to simply diagnose DSPN and estimating the severity of DSPN is also a formidable challenge for clinicians. Not all patient present typical signs and symptoms of neuropathy and clinical sensory examinations are inadequate for accurate diagnosis and follow-up (Dyck et al., 2010). Previously, abnormalities in nerve conduction were earlier thought to be a result of advance diabetes and be appears to the first objective and quantitative indication of DSPN (Veves et al., 1991; England et al., 2009). Recent studies have assessed the criteria of nerve conduction abnormalities in DSPN more accurately and provided a method for estimating the severity of DSPN (Dyck et al., 2011a). Several previous electrophysiologic studies have used limited evaluations without adequate reference values in nerve conduction studies (NCS) from themselves laboratory, or only identified the absence or presence of DSPN without quantification of electrophysiological severity (Rendell et al., 1989; Ziegler et al., 2004; Hotta et al., 2012; Zhu and Yang, 2018). In the Rochester Diabetic Neuropathy Study (Dyck et al., 1997, 2011b), Dyck et al. concluded that composite sum scores of representative attributes of nerve conduction have been shown to be useful for estimating the severity of polyneuropathies, rather than just using one or two nerve conduction abnormalities.

Diabetes Mellitus refers to a constellation of abnormalities, mainly characterized by hyperglycemia along with disturbance in carbohydrate, fat and protein metabolism (Mohapatra and Damodar, 2016). Glycemic variability (GV) is a term used to describe impairment of glycemic control. Multiple measures of GV have been suggested as potential predictors for DSPN (Xu et al., 2014; Jin et al., 2016; Yang et al., 2017), and glycemic control influences nerve conduction abnormalities (Dcct Research Group, 1995). Long-term GV assessment using HbA1c variability is significantly associated with the presence of DSPN (Su et al., 2018). Besides impairment of glycemic control, there are several cardio-metabolic parameters (e.g., lipid profile and renal parameters) can also contribute to the progression of DSPN (Mohapatra and Damodar, 2016).

To date, there is paucity of information that focuses on estimating the overall severity of DSPN using composite scores and investigating its relationship between chronic glycemic impairment and different cardio-metabolic parameters. Exploring the role of the chronic glycemic variability on the subsequent severity of DSPN and developing therapeutic strategies that can prevent or improve the severity of DSPN would be beneficial in patients with diabetes. Therefore, potential risk factors need to be delineated to determine patients who are most appropriate for aggressive treatment. In this study, we tested the hypotheses that chronic glycemic variability can influence on the severity of DSPN, and there is a close relationship between HbA1C variability and peripheral nerve amplitudes and velocities, in patients with type 2 diabetes. The successful translation of these approaches to the clinics offers the promise of reducing microvascular complications and improving quality of life in patients with type 2 diabetes.

## Patients And Methods

### Patients

A total of 250 patients (age range, 29–90 years) with type 2 diabetes who visited the outpatient diabetic clinic at Kaohsiung Chang Gung Memorial Hospital in Taiwan were recruited. Exclusion criteria were (1) no follow-up within the first 6 months of outpatient clinic visit and (2) the numbers of HbA1c measurements are < 4. Thus, we enrolled 223 participants were enrolled in the study. The study was approved by the Ethics Committee of Chang Gung Memorial Hospital Institutional Review Board (CGMH IRB 96-1361B, 201701243B0, and 201800388B0).

### Baseline Clinical and Laboratory Measurements

All patients underwent complete neurologic and physical examinations upon enrollment and at subsequent follow-up sessions at the outpatient clinic. A detailed medical history regarding the prior use of medications was obtained from patients and their families using standardized questions. Demographic data, including age, sex, duration of diabetes (years), body height and weight, waist circumstance, systolic blood pressure (SBP) and diastolic blood pressure (DBP) during autonomic function testing, microvascular complication of diabetes (retinopathy and nephropathy), and laboratory parameters were obtained at baseline. DSPN was diagnosed in patients who not only had neurological symptoms/signs and abnormalities in nerve conduction evaluations but also fulfilled the diagnostic criteria of DSPN based on the clinical research Report of the American Academy of Neurology (Feldman et al., 1994; England et al., 2005, 2009). Diabetic retinopathy (DR) was classified as one of the following three stages: stage 0, no apparent retinopathy; stage 1, non-proliferative DR; and stage 2, proliferative DR (Wilkinson et al., 2003). The abbreviated Modification of Diet in Renal Disease study formula recalibrated for patients was used to estimate glomerular filtration rate (eGFR):eGFR = 186 × [serum creatinine (mmol/L) × 0.011]^−1.154^ × [age]^−0.203^ × [0.742 if female] × 1.233, where 1.233 is the adjusting coefficient for Chinese patients (Ma et al., 2006). Albuminuria was assessed by measuring the urinary albumin-to-creatinine ratio (UACR) in spot urine.

Vascular risk factors, including hypertension, defined as SBP >140 mmHg and/or DBP >90 mmHg or being under treatment with anti-hypertensive agents; and dyslipidemia, defined as total cholesterol level >200 mg/dL, triglyceride level >180 mg/dL, or having a prescription for lipid-lowering medication at the time of the study. Metabolic syndrome was defined as the presence of at least two of the following criteria: (1) waist circumstance >90 cm for men and >80 cm for women; (2) serum triglyceride level ≥150 mg/dL or having a prescription for elevated triglyceride levels; (3) serum high-density lipoprotein cholesterol (HDL-C) < 40 mg/dL in men and < 50 mg/dL in women or having a prescription for low HDL-C levels; and (4) elevated blood pressure (SBP ≥130 mmHg, and/or DBP ≥85 mmHg, or a previous diagnosis of hypertension).

### Assessment of Glycemic Variability

For each patient, the intrapersonal mean, SD, and coefficient of variation (CV = SD-HbA1c/[0.1 × mean HbA1c]) of HbA1c were calculated using all measurements obtained 3 years prior to the study start date. The SD-HbA1c and CV were considered as measures of glycemic variability and normalized glycemic variability, respectively. The number of individual visits could influence SD-HbA1c values (with fewer visits likely to artificially inflate SD), we defined the adjusted SD of HbA1c as the SD of HbA1c divided by [*n*/(*n* – 1)]^0.5^, where n is the number of HbA1c measurements) to minimize any effect of different numbers of HbA1c measurements on the values calculated (Kilpatrick et al., 2008). Patients were divided into quartiles using each of these indices or mean HbA1c.

### Assessment and Scoring of Nerve Conduction Studies

The NCS were performed using Nicolet Viking machines. All measurements were recorded using standard laboratory methods. All studies included surface recording and stimulation. The belly-tendon montage was used and supra-maximal stimulation was applied. All data obtained were compared with reference values from our laboratory (Huang et al., 2009). The sensory and motor nerves of the non-dominant side were tested. The NCS recordings were performed according to a previously published method (Huang et al., 2009). We performed motor nerve studies, including the median, ulnar, tibial and peroneal nerves, and sensory nerve studies, including the median, ulnar and sural nerves. The following attributes were measured: distal latency, amplitude, and nerve conduction velocity (NCV).

To improve assessment of composite scores of representative attributes of nerve conduction in the Rochester Diabetic Neuropathy Study (Dyck et al., 1997, 2011b), Dyck et al. constructed modified composite scores of nerve conduction as the severity of peripheral neuropathy, according to the transthyretin amyloid polyneuropathy trials (Suanprasert et al., 2014). The modified composite scores for use in DSPN was the sum of the five normal deviates of nerve conduction. The sum of five nerve conduction normal deviate scores consisted of peroneal nerve compound muscle action potential (CMAP) amplitude, tibial CMAP amplitude, ulnar CMAP amplitude, sural sensory nerve action potential (SNAP) amplitude, and ulnar SNAP amplitude. By using only amplitudes of motor and sensory nerve conduction, all attributes of nerve conduction were more assessable because conduction velocities and distal latencies were too frequently unmeasurable if peroneal and tibial CMAPs were 0 (Dyck et al., 1997, 2011b; Suanprasert et al., 2014). These values were transformed to normal deviates from percentile values by correcting for age, gender, height, or weight as based on our previous study (Huang et al., 2009). Additionally, these percentile values were expressed as points from obtained percentile values (for example, N5th = 0 points; ≤ 5th–N1st = 1 point and ≤ 1st = 2 points; and similarly when the abnormality is in the upper tail of the normal distribution). The five attributes of nerve conduction provided a scale from 0 to 10 points.

### Statistical Analysis

Data are expressed as mean ± SD or median (interquartile range). Categorical variables were compared using chi-square or Fisher's exact tests. Continuous variables that were not normally distributed were logarithmically transformed to improve normality prior to analysis. Three separate statistical analyses were performed. First, trends between more than two groups were analyzed using linear polynomial contrasts ANOVA for normally distributed variables. Second, correlation analysis was used to evaluate the relationship between composite scores of nerve conduction and variables including age, diabetes duration, body mass index (BMI), waist circumstance, systolic and diastolic blood pressure, and peripheral blood studies for vascular risks. Third, two stepwise models of multiple linear regression analysis were performed to evaluate the influence of independent variables on the mean composite scores of nerve conduction. Factors that were significantly correlated with the mean composite scores of nerve conduction were assessed using the model 1 multiple linear regression analysis. Subsequently, results from model 1 were further analyzed using the model 2 multiple linear regression analysis. Fourth, the changes in composite scores of nerve conduction and HbA1c variability and cardio-metabolic parameters during follow-up were defined as follows: delta = (data after the follow-up) – (data at the beginning). A correlation analysis was used to determine the relationship between changes in HbA1c variability and those in composite scores of nerve conduction after controlling with the follow-up duration (months). All statistical analyses were conducted using the SAS software package, version 9.1 (2002, SAS Statistical Institute, Cary, North Carolina).

## Results

### General Characteristics of Patients With Diabetes

The 223 patients with diabetes in this study included 78 women (age range, 35–82 years; mean age, 63.4 years) and 145 men (age range, 29–90 years; mean age, 62.3 years). Patient characteristics and complication rates at last assessment are presented in Tables 1, 2, stratified by ascending quartiles of SD-HbA1c. Those with higher SD-HbA1c values had higher body weight (*P* = 0.04) and body mass index (*P* = 0.004), higher mean and index HbA1c values (*P* < 0.0001 and *P* < 0.0001, respectively), higher triglyceride and uric acid levels (*P* < 0.001 and *P* < 0.0001, respectively), higher urinary albumin excretion (mg/day), higher albumin-creatinine ratio (mg/mg) (*P* = 0.008 and *P* = 0.002, respectively), lower eGFR (*P* < 0.0001), higher prevalence of hypertension and metabolic syndrome as the underlying disease (*P* = 0.03 and 0.001), and a higher proportion of insulin therapy as treatment (*P* = 0.001).

**Table 1:** Characteristics of patients with Type 2 diabetes stratified by ascending quartiles of glycemic variability (HbA1c SD).

	**1st (*n* = 56)**	**2nd (*n* = 55)**	**3rd (*n* = 57)**	**4th (*n* = 55)**	**Total**	***P-*value for trend**
**CHARACTERISTICS**						
Age (year)	62.8 ± 10.5	63.0 ± 9.2	64.0 ± 8.3	60.9 ± 10.5	62.7 ± 9.6	0.38
Sex (female/male)	37/19	34/21	37/20	37/18	37/19	0.94
Diabetes duration (year)	9.6 ± 8.7	11.9 ± 6.8	11.6 ± 7.8	13.0 ± 7.5	11.5 ± 7.7	0.11
Height (cm)	163.2 ± 8.2	161.4 ± 8.0	160.8 ± 7.8	162.8 ± 8.0	162.0 ± 8.0	0.36
Body weight (Kg)	65.9 ± 11.4	68.3 ± 12.8	70.0 ± 12.5	72.5 ± 13.5	69.2 ± 12.7	0.04^*^
Body mass index	24.7 ± 3.4	25.9 ± 3.7	26.7 ± 4.4	27.3 ± 3.8	26.1 ± 3.9	0.004^*^
Waist circumstance (cm)	89.7 ± 10.9	92.4 ± 9.4	93.7 ± 10.2	96.0 ± 11.7	93.1 ± 10.7	0.11
SBP (mmHg)	137.2 ± 21.7	136.4 ± 19.2	143.9 ± 18.2	140.5 ± 17.1	139.5 ± 15.2	0.15
DBP(mmHg)	74.5 ± 10.1	72.7 ± 11.6	76.1 ± 10.5	76.0 ± 10.8	74.8 ± 10.8	0.31
**BASELINE UNDERLYING DISEASE**						
Hypertension (%)	41 (73.2%)	51 (92.7%)	50 (87.7%)	48 (87.3%)	190 (85.2%)	0.03^*^
Coronary heart disease (%)	6 (10.7%)	9 (16.3%)	15 (26.3%)	7 (12.7%)	37 (16.6%)	0.12
Ischemic stroke (%)	9 (16.1%)	11 (20%)	13 (22.8%)	9 (16.3%)	42 (18.8%)	0.77
Retinopathy (%)	11 (19.6%)	11 (20%)	17 (29.8%)	19 (34.5%)	58 (26%)	0.28
Metabolic syndrome (%)	28 (50%)	44 (80%)	46 (80.7%)	40 (72.7%)	158 (70.8%)	0.001^*^
**TYPE OF DIABETES TREATMENT**						
Insulin + OHA	1	0	0	0	1	
OHA only	52	42	36	31	161	0.001^*^
Insulin only	2	12	18	21	53	
No treatment	1	1	3	3	8	
**OTHER CONCOMITANT MEDICATIONS**						
ACE inhibitor or ARB	38	44	42	42	166	0.51
Beta-blocker	13	21	24	14	72	0.08
Calcium channel blocker	19	25	26	17	87	0.25
Diuretics	18	30	31	29	108	0.046^*^
Antiplatelet medications	42	37	38	35	152	0.61
Lipid-lowering medications	38	43	48	45	174	0.16

Data are presented as means ± standard deviations or n (%). n, number of cases; SBP, systolic blood pressure; DBP, diastolic blood pressure; OHA, oral hypoglycemic agent; ACE, angiotensin-converting enzyme; ARB, angiotensin II receptor blocker.

* *p < 0.05*.

Bold values indicates statistical significance.

**Table 2:** Laboratory test findings of patients with Type 2 diabetes stratified by ascending quartiles of glycemic variability (HbA SD).

	**1st (*n* = 56)**	**2nd (*n* = 55)**	**3rd (*n* = 57)**	**4th (*n* = 55)**	**Total**	***P-*value for trend**
**LABORATORY TEST FINDINGS**						
Total cholesterol(mmol/L)	156.4 ± 24.3	156.8 ± 30.0	158.6 ± 32.1	153.1 ± 32.3	156.3 ± 29.7	0.81
Triglyceride(mmol/L)	108.1 ± 66.1	151.3 ± 86.4	127.5 ± 60.2	168.5 ± 95.5	138.81 ± 81.0	<0.001^**^
HDL-C (mmol/L)	55.6 ± 11.9	51.7 ± 13.5	50.3 ± 12.0	49.8 ± 17.8	51.9 ± 14.1	0.12
LDL-C (mmol/L)	79.2 ± 22.3	75.1 ± 26.9	82.8 ± 29.6	72.0 ± 27.6	77.3 ± 26.9	0.16
UA (mmol/L)	6.3 ± 1.7	7.1 ± 1.5	7.2 ± 2.0	7.5 ± 2.2	7.1 ± 1.9	0.007^*^
hs-CRP (mmol/L)	2.7 ± 1.4	1.8 ± 1.6	2.5 ± 1.6	3.5 ± 2.7	2.6 ± 1.1	0.76
Index HbA (%)	6.6 ± 0.6	7.2 ± 0.8	7.5 ± 0.9	7.6 ± 1.1	7.2 ± 1.0	<0.0001^**^
Mean HbA (%)	6.6 ± 0.6	7.4 ± 0.8	7.8 ± 0.8	8.3 ± 1.0	7.5 ± 1.0	<0.0001^**^
CV HbA	4.2 ± 1.2	7.2 ± 1.2	10.1 ± 1.4	17.0 ± 6.0	9.6 ± 5.7	<0.0001^**^
Urinary albumin excretion (mg/day)	5.9 ± 12.1	20.0 ± 41.1	49.9 ± 39.4	60.9 ± 39.0	34.5 ± 14.1	0.008^*^
Albumin-creatinine ratio (mg/mg)	85.4 ± 15.6	375.7 ± 115.0	464.6 ± 92.7	1026.3 ± 176.8	486.0 ± 116.5	0.002^*^
eGFR (mL/min/)	79.5 ± 34.7	61.3 ± 26.1	59.6 ± 28.5	57.9 ± 30.2	64.6 ± 34.7	<0.0001^**^

Data are presented as means ± standard deviations or n (%). n, number of cases; HDL-C, high-density lipoprotein cholesterol; LDL-C, low-density lipoprotein cholesterol; UA, uric acid; hsCRP, high-sensitive C-reactive protein; HbA, glycohemoglobin; eGFR, estimated glomerular filtration rate; CV, coefficient of variation.

* p < 0.05,

** *p < 0.0001*.

Bold values indicates statistical significance.

### The Relationship Between HbA1c SD and Nerve Conduction Study

The NCS in patients with type 2 diabetes, stratified by ascending quartiles of GV (SD-HbA1c), are listed in Table 3. Patients with higher SD-HbA1c had lower compound muscle action potential (CMAP) values in the ulnar (*P* = 0.001), peroneal (*P* = 0.04), and tibial (*P* = 0.001) nerves; and lower sensory nerve action potential (SNAP) values in the median (*P* < 0.0001), ulnar (*P* < 0.0001), and sural (*P* < 0.0001) nerves. Patients with higher SD-HbA1c had lower motor nerve conduction velocity (MNCV) values in median (*P* < 0.0001), ulnar (*P* = 0.02), peroneal (*P* = 0.02), and tibial (*P* = 0.001) nerves; and lower sensory nerve conduction velocity (SNCV) values in the sural nerve (*P* = 0.04). Furthermore, patients with higher SD-HbA1c also had higher composite score of low extremities (*P* = 0.026).

**Table 3:** Nerve conduction study in patients with Type 2 diabetes stratified by ascending quartiles of glycemic variability (HbA SD).

	**1st (*n* = 56)**	**2nd (*n* = 55)**	**3rd (*n* = 57)**	**4th (*n* = 55)**	***P*-value for trend**
**MEDIAN NERVE, MOTOR**					
DML	4.2 ± 0.8	4.8 ± 1.2	4.3 ± 1.1	4.7 ± 1.3	0.02^*^
CMAP	8.6 ± 2.8	7.8 ± 2.3	8.2 ± 2.4	7.8 ± 2.8	0.25
MNCV	53.2 ± 4.8	50.9 ± 4.8	51.3 ± 4.1	49.0 ± 4.9	<0.0001^**^
**ULNAR NERVE, MOTOR**					
DML	2.9 ± 0.4	3.0 ± 0.5	3.1 ± 0.8	3.2 ± 0.7	0.04^*^
CMAP	9.3 ± 2.6	8.6 ± 2.4	8.1 ± 2.8	7.1 ± 3.3	0.001^*^
MNCV	52.3 ± 6.9	49.1 ± 7.5	49.6 ± 7.1	48.0 ± 7.4	0.02^*^
**PERONEAL NERVE**					
DML	4.1 ± 0.9	4.3 ± 1.0	4.3 ± 0.8	4.4 ± 1.1	0.7
CMAP	4.1 ± 2.5	3.2 ± 2.5	3.3 ± 2.2	2.8 ± 2.3	0.04^*^
MNCV	43.6 ± 5.5	41.5 ± 5.8	42.0 ± 5.4	40.2 ± 5.2	0.02^*^
**TIBIAL NERVE**					
DML	4.3 ± 0.9	4.3 ± 0.9	4.4 ± 1.2	4.4 ± 0.8	0.9
CMAP	10.6 ± 5.4	8.7 ± 4.6	8.1 ± 4.5	6.7 ± 4.9	0.001^*^
MNCV	43.3 ± 5.0	41.5 ± 4.9	40.8 ± 4.8	39.4 ± 4.9	0.001^*^
**MEDIAN NERVE, SENSORY**					
Latency	3.2 ± 1.3	3.5 ± 0.9	3.1 ± 0.7	3.4 ± 0.9	0.24
SNAP	29.4 ± 17.4	20.7 ± 14.4	23.1 ± 14.1	16.7 ± 14.6	<0.0001^**^
SNCV	46.5 ± 9.6	42.7 ± 9.9	46.2 ± 8.4	43.3 ± 10.8	0.09
**ULNAR NERVE, SENSORY**					
Latency	2.3 ± 0.4	2.4 ± 0.3	2.5 ± 0.4	2.4 ± 0.5	0.3
SNAP	27.0 ± 16.7	19.3 ± 13.1	16.7 ± 13.9	12.4 ± 12.1	<0.0001^**^
SNCV	50.9 ± 9.7	61.0 ± 43.1	48.5 ± 6.7	49.5 ± 7.7	0.3
**SURAL NERVE**					
Latency	2.9 ± 0.5	2.9 ± 0.6	3.1 ± 0.4	2.9 ± 0.9	0.24
SNAP	9.4 ± 6.1	8.2 ± 6.6	5.3 ± 4.9	4.6 ± 3.6	<0.0001^**^
SNCV	49.4 ± 6.7	47.2 ± 6.2	45.8 ± 6.5	46.2 ± 6.7	0.04^*^
Composite score of low extremities	4.8 ± 4.2	5.9 ± 4.4	6.3 ± 4.4	7.4 ± 4.8	0.026^*^

Data are presented as means ± standard deviations or n (%). n, number of cases; DML, distal motor latency; CMAP, compound muscles action potential; MNCV, motor nerve conduction velocity; SNAP, sensory nerve action potential; SNCV, sensory nerve conduction velocity.

* p < 0.05,

** *p < 0.0001*.

Bold values indicates statistical significance.

### Effect of Glycemic Variability and Other Vascular Risk Factors on Composite Scores of Nerve Conduction in Patients With Type 2 Diabetes

The correlation analysis used to test the influence of glycemic variability and other vascular risk factors on composite scores of nerve conduction are listed in Table 4. The statistical results (correlation coefficient, *P*-value) were as follows: age (year; *r* = 0.177, *P* = 0.008), diabetes duration (year; *r* = 3.67, *P* < 0.0001), systolic blood pressure (SBP, mmHg; *r* = 0.15, *P* = 0.025), uric acid (UA, mmol/L; *r* = 0.253, *P* < 0.0001), hs-CRP (mmol/L; *r* = 0.154, *P* = 0.023), mean HbA1c (%; *r* = 0.157, *P* = 0.019), CV HbA1c (*r* = 0.304, *P* < 0.0001), SD HbA1c (*r* = 0.304, *P* < 0.0001), and eGFR (mL/min/1.73 m^2^) (*r* = −0.529, *P* < 0.0001). Furthermore, correlation analysis was used to test the influence of GV on composite scores after controlling for age, diabetes duration, eGFR, and mean HbA1c (%; *r* = 0.095, *P* = 0.161) and SD-HbA1c (*r* = 0.209, *P* = 0.002).

**Table 4:** Correlation analysis of composite scores of nerve conduction in patients with type 2 diabetes.

**Variables**	**Composite scores of nerve conduction**	
	***r***	***P*-value**
Age (year)	0.177	0.008^*^
Diabetes duration (year)	0.367	<0.0001^**^
Body mass index	0.025	0.714
Waist circumstance (cm)	0.129	0.125
SBP (mmHg)	0.15	0.025^*^
DBP(mmHg)	−0.01	0.866
Total cholesterol(mmol/L)	−0.061	0.37
Triglyceride(mmol/L)	−0.03	0.896
HDL-C (mmol/L)	−0.055	0.417
LDL-C (mmol/L)	−0.099	0.889
UA (mmol/L)	0.253	<0.0001^**^
hs-CRP (mmol/L)	0.154	0.023^*^
Index HbA (%)	0.062	0.305
Mean HbA (%)	0.157	0.019^*^
CV HbA	0.304	<0.0001^**^
SD HbA	0.304	<0.0001^**^
eGFR (mL/min/)	−0.529	<0.0001^**^

r, correlation coefficient.

* p < 0.05,

** *p < 0.0001. SBP, systolic blood pressure; DBP, diastolic blood pressure; OHA, oral hypoglycemic agent; ACE, angiotensin-converting enzyme; ARB, angiotensin II receptor blocker; HDL-C, high-density lipoprotein cholesterol; LDL-C, low-density lipoprotein cholesterol; UA, uric acid; hsCRP, high-sensitive C-reactive protein; HbA, glycohemoglobin; eGFR, estimated glomerular filtration rate; CV, coefficient of variation. Bold values indicates statistical significance.*.

### Clinical Factors Are Significantly Associated With Composite Scores of Nerve Conduction in Patients With Diabetes

The effects of the variables on composite scores of nerve conduction in patients with type 2 diabetes relating to the correlation analysis are listed in Table 5. Following this, two series of statistical analysis were carried out to decipher the relationships between the augmented composite scores of nerve conduction in patients with diabetes, GV, and other vascular risk factors. Our first analysis revealed that age, diabetes duration, SBP, UA, hs-CRP, mean HbA1c, SD-HbA1c, and eGFR were correlated with composite scores of nerve conduction (Table 4). Next, we employed multiple linear regression analysis to identify variables of the crucial determinant that underlie the augmented composite scores of nerve conduction in patients with diabetes. Results from the model 1 analysis (Table 5) revealed that diabetes duration, SD-HbA1c, and eGFR were significantly associated with mean composite scores of nerve conduction while there was no association with age, SBP, UA, hs-CRP, and mean HbA1c. Therefore, we included diabetes duration, SD-HbA1c, and eGFR in the Model 2 stepwise regression analysis and found that diabetes duration, SD-HbA1c, and eGFR were independently associated with mean composite scores of nerve conduction in the patients with type 2 diabetes.

**Table 5:** Effects of the variables on composite scores of nerve conduction in patients with type 2 diabetes according to correlation analysis.

	**Model 1**			**Model 2**		
	**Regression coefficient**	**Standard error**	***P*-value**	**Regression coefficient**	**Standard error**	***P*-value**
Constant	5.12	2.65	0.054	5.68	0.69	<0.0001^**^
SD HbA	1.25	0.5	0.014^*^	1.33	0.43	0.002^*^
eGFR	−0.042	0.007	<0.0001^**^	−0.044	0.006	<0.0001^**^
Diabetes duration	0.079	0.027	0.003^*^	0.079	0.025	0.002^*^
Age	0.01	0.021	0.625			
Mean HbA	−0.015	0.208	0.941			
Uric acid	−0.009	0.112	0.933			
hs-CRP	0.026	0.024	0.278			
Systolic blood pressure	< 0.0001	0.01	0.97			

Regression coefficient for each individual variable. eGFR, estimated glomerular filtration rate; BMI, body mass index.

* p < 0.05,

** *p < 0.0001*.

Bold values indicates statistical significance.

### Effects of Changes of Glycemic Variability and Cardio-Metabolic Parameters on the Changes of Composite Scores

Among the 223 cases, 21 patients died during the follow-up period. The mean follow-up period of the remaining 202 patients was 67.2 months. The changes in parameters including composite score of nerve conduction and glycemic variability during the follow-up period were determined as follows: data in the final follow-up examination—baseline data. The correlation analysis was used to determine the relationship between the changes in mean, CV and SD HbA1c and those in composite scores after controlling with the NCS follow-up duration (months) (Table 6). The statistically significant results are presented as follows. CV HbA1c and composite scores: *r* = 0.555, *P* = 0.049.

**Table 6:** Effects of changes of glycemic variability and cardio-metabolic parameters on the changes of composite scores.

	Δ**Composite scores of nerve conduction^§^**	
	***r***	***P*-value**
ΔIndex HbA (%)	−0.165	0.543
ΔMean HbA	0.069	0.815
ΔCV HbA	0.555	0.049^*^
ΔSD HbA	0.532	0.061
Δ Total cholesterol(mmol/L)	0.258	0.394
ΔTriglyceride(mmol/L)	−0.3	0.277
ΔHDL-C (mmol/L)	0.119	0.7
ΔLDL-C (mmol/L)	0.263	0.364
ΔUA (mmol/L)	0.491	0.217
ΔeGFR (mL/min/)	0.094	0.738

Δ, Mean changes during follow-up period (Final follow-up minus baseline data).

§ The statistical analysis was used by partial correlation after controlling with follow-up duration (months). HDL-C, high-density lipoprotein cholesterol; LDL-C, low-density lipoprotein cholesterol; UA, uric acid; HbA, glycohemoglobin; eGFR, estimated glomerular filtration rate; CV, coefficient of variation;

* *p < 0.05; r, correlation coefficient*.

Bold values indicates statistical significance.

## Discussion

### Major Findings of Our Study

To date, this is the first study to confirm the hypothesis that HbA1C variability is strongly associated with composite scores of nerve conduction, which are indicative of the degree of severity of DSPN, in patients with type 2 diabetes. We examined the severity of somatic nerve dysfunction by creating a composite score to assess different stages of chronic glycemic impairment, and correlated this score with different cardio-metabolic parameters to obtain four major findings.

First, patients with higher SD-HbA1c values had increased body weight and body mass index, higher mean and index HbA1c values, higher triglyceride and uric acid levels, higher urinary albumin excretion and albumin-creatinine ratios, an increased proportion of insulin therapy and prevalence of hypertension and metabolic syndrome, and lower eGFR. Second, patients with higher SD-HbA1c values had lower CMAP, SNAP, and MNCV on tested nerves and lower SNCV on sural nerve. Third, patients with higher SD-HbA1c values also had higher composite score of low extremities. Finally, multiple linear regression analysis revealed that diabetes duration, SD-HbA1c, and eGFR were independently associated with mean composite scores of nerve conduction.

### The Importance of Glycemic Variability in Diabetic Complications

Aggressive control of blood glucose is pivotal for patients with type 2 diabetes, and can prevent microvascular (nephropathy, retinopathy and neuropathy) and macrovascular (coronary artery diseases, stroke, and peripheral artery diseases) complications (Brownlee, 2001; Brownlee and Hirsch, 2006; Siegelaar et al., 2010). GV indicated that patients with similar mean glucose or HbA1c values can show markedly different daily glucose profiles, with differences in the number and duration of glucose excursions (Brownlee, 2001; Brownlee and Hirsch, 2006; Siegelaar et al., 2010). Fluctuating or constant high glucose levels can induce oxidative stress, overproduction of reactive oxygen species, and contribute to endothelial dysfunction. Finally, the serial process results in diabetic complications (Brownlee, 2001; Brownlee and Hirsch, 2006; Siegelaar et al., 2010). Further, there is some evidence that long-term GV might be related to microvascular complications in type 2 diabetes (Hirakawa et al., 2014; Jin et al., 2016; Wei et al., 2016; Su et al., 2018). Our study demonstrated that GV and urinary albumin excretion were positively correlated, and GV was negatively correlated with eGFR, amplitude (CMAP and SNAP), and conduction velocity (MNCV and SNCV) of the nerves in the low extremities.

### Glycemic Variability and Other Potential Risk Factors Associated With the Occurrence of DPN

Several well-known risk factors are associated with the occurrence of DPN (Dyck et al., 1999; Tesfaye et al., 2005). One clinical study demonstrated that the lower prevalence of polyneuropathy in those with duration of DM < 5 years and highest in those with duration of DM >15 years (Nisar et al., 2015). Both chronic poor glycemic control and variability are some of the most important risk factors for DSPN. Further, each 1% increment in HbA1c is associated with an approximately 10–15% increase in diabetic neuropathy frequency (Bansal et al., 2006). One recent study from China has also demonstrated that increased HbA1c variability adds to poor chronic glycemic control and is closely associated with diabetic neuropathy in patients with type 2 diabetes (Su et al., 2018). Lower eGFR and higher UACR may be associated with increased risk of DPN even though normal or mildly abnormal UACR and eGFR have already been found to be predictive factors of diabetic neuropathy (Zhang et al., 2017).

### Glycemic Variability and Other Risk Factors Associated With the Severity of DSPN

The above-mentioned risk factors for DSPN that were identified may provide important clues to etiologies, or merely reflect chance associations. If a particular risk factor is implicated in the pathophysiology of DSPN when the same risk factor is found consistently to severity of the DSPN, then there is a plausible mechanistic link between risk factor and disease progression. Most previous studies have demonstrated that chronic glycemic impairment (HbA1C value) contribute to the severity of DPN (Tkac and Bril, 1998; Dyck et al., 1999; Perkins et al., 2001); however, only one study has focused on the relationship between GV and occurrence of DPN [9]. To the best of our knowledge, no animal or clinical study has investigated the impact of GV on the severity of DPN in type 2 diabetes. One longitudinal study of 264 individuals with diabetes, including type 1 and type 2 diabetes, demonstrated that diabetic micro-vessel disease, chronic hyperglycemic exposure (HbA1c value), and type of diabetes are associated with a composite score of the severity of DPN in a 7-year follow-up study (Dyck et al., 1999). Another cross-sectional study enrolled 97 patients with type 1 or type 2 diabetes with DPN and demonstrated that the severity of DPN, expressed by the sum of nerve conduction velocities and distal amplitude scores, was significantly related to glycemic control (HbA1c value) (Tkac and Bril, 1998). Another observational cohort study enrolled 89 patients with type 1 and type 2 diabetes, ascertained from a large therapeutic randomized clinical trial, and found that the morphological severity of DPN, expressed as the myelinated fiber density in sural nerve biopsies, was significantly associated with glycemic control (HbA1c value) (Perkins et al., 2001). Further, one study enrolled 189 patients with type 1 and/or type 2 diabetes with or without DPN and/or cardiovascular autonomic neuropathy (CAN) and 85 healthy controls, and demonstrated that the severity of DPN, expressed using the Neuropathy Impairment Score of the Lower Limbs, was related to oxidative stress, diabetes duration, and triglyceride levels (Ziegler et al., 2004). The discrepancy between these studies and our study may be attributed to different methodologies (for example, the inclusion of patients with type 1 or type 2 diabetes, patients with or without DPN, a cross-sectional or longitudinal study, and electrophysiological or morphological severity of DPN), diabetic duration, good or poor glycemic control, and statistical methods. While it is cross-sectional observation study, our study confirmed that HbA1c variability, diabetes duration, and eGFR were strongly associated with composite scores of nerve conduction, which may indicate the degree of DSPN severity in patients with type 2 diabetes. Therefore, the therapeutic regimens used to treat DPN should be used to aggressively control blood glucose to an acceptable range (Amthor et al., 1994) and avoid fluctuations in blood glucose to help to prevent further nerve damage or an exacerbation of symptoms (Bansal et al., 2006).

### Study Limitations

This study has several limitations. First, we excluded patients who were lost to follow-up within the first 6 months from the outpatient clinic and the numbers of HbA1c measurements are <4. Thus, there is uncertainty in assessing the role of chronic glycemic impairment and other cardio-metabolic parameters in unselected type 2 diabetes. Second, while there was a positive association between HbA1c variability and composite scores of nerve conduction in this observational study, it is unclear whether the role of the association is causal. A prospective longitudinal study is necessary to evaluate the efficiency of composite scores of nerve conduction as a scale of severity in clinical follow up. Third, we only used composite scores of nerve conduction as the quantitative approaches of severity of DSPN. Because only large fiber function was assessed, it may not account for at all the variability in severity found in DSPN, including any statistical bias. Other quantitative approaches to assess the severity of DPN, such as morphological severity and/or Neuropathy Impairment Score of the Lower Limbs, should be taken into account for future studies. Fourth, our study only included a Chinese population with type 2 diabetes. Finally, although the sample size was small, the number of variables that we considered for the multiple linear regression analysis was also small. Furthermore, based on the stepwise procedures, only three variables were selected as variables that were important in predicting the composite score. Therefore, the maximum likelihood estimates of the coefficients are valid in this analysis.

## Conclusion

Based on our results, the severity of neuropathy assessed by detailed electro-physiological testing was clearly associated by high HbA1c variability. As HbA1c variability is considered a prognostic factor, aggressively control blood glucose to an acceptable range and avoid fluctuations in blood glucose by more individualized treatment to prevent further nerve damage.

## Availability of Data and Materials

The data from this study can be acquired from the corresponding author upon reasonable request.

## Author Contributions

Y-RL participated in the design of the study and drafted the manuscript. W-CC, C-CH, N-WT, H-CW, W-CL, B-CC, Y-JS, C-MS, and S-YH participated in the sequence alignment and clinical evaluation of patients. C-CH performed the statistical analysis. C-HL conceived the study, and participated in its design and coordination, and helped draft the manuscript. All authors read and approved the final manuscript.

### Conflict of Interest Statement

The authors declare that the research was conducted in the absence of any commercial or financial relationships that could be construed as a potential conflict of interest.

## Abbreviations

SD: standard deviation
eGFR: estimate glomerular filtration rate
DPN: diabetic peripheral neuropathy
DSPN: distal symmetric sensorimotor polyneuropathy
GV: glycemic variability
SBP: systolic blood pressure
DBP: diastolic blood pressure
DR: diabetic retinopathy
UACR: urinary albumin-to-creatinine ratio
NCS: nerve conduction studies
NCV: nerve conduction velocity
BMI: body mass index
CMAP: compound muscle action potential
SNAP: sensory nerve action potential
MNCV: motor nerve conduction velocity
SNCV: sensory nerve conduction velocity
CAN: cardiovascular autonomic neuropathy.
